# Is it time for biocatalysis in fragment-based drug discovery?

**DOI:** 10.1039/d0sc04103c

**Published:** 2020-10-07

**Authors:** Jeremy I. Ramsden, Sebastian C. Cosgrove, Nicholas J. Turner

**Affiliations:** Department of Chemistry, Manchester Institute of Biotechnology, University of Manchester 131 Princess Street Manchester M1 7DN UK Nicholas.turner@manchester.ac.uk; Future Biomanufacturing Research Hub, Manchester Institute of Biotechnology, University of Manchester 131 Princess Street Manchester M1 7DN UK Sebastian.cosgrove@manchester.ac.uk; School of Chemical and Physical Science, Lennard-Jones Laboratories, Keele University Staffordshire ST5 5BG UK

## Abstract

The use of biocatalysts for fragment-based drug discovery has yet to be fully investigated, despite the promise enzymes hold for the synthesis of poly-functional, non-protected small molecules. Here we analyze products of the biocatalysis literature to demonstrate the potential for not only fragment generation, but also the enzyme-mediated elaboration of these fragments. Our analysis demonstrates that biocatalytic products can readily populate 3D chemical space, offering diverse catalytic approaches to help generate new, bioactive molecules.

## Abstract 2

This perspective discusses how biocatalysis could play an important role in the future fragment-based drug discovery.

## Graphical abstract

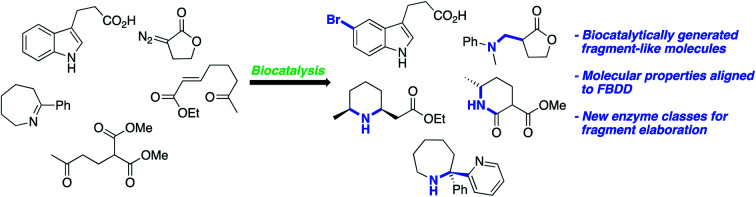

## Introduction

Synthetic pharmaceuticals are dominated by sp^2^-rich molecules, and a small subset of functionalities and backbone structures.^1–3^ This has been attributed to the available methodologies, with transition-metal catalysed cross-coupling reactions featuring strongly in the way that compounds are made.^4^ In particular, the discovery of Pd-catalysed methods in the late 1970s, namely the Suzuki, Negishi, Stille and Heck reactions, the further development of them and other reactions such as the Buchwald–Hartwig reaction, and additionally amide bond formation, has delivered a rich toolbox of sp^2^ cross-coupling reactions,^5,6^ and consequently sp^2^-rich products. Despite the consistent, and successful, synthesis of generally 2D molecules it is well-documented that sp^3^-rich molecules have a higher success rate as drug candidates.^7,8^ Therefore, new methodologies that produce diverse sets of 3D molecules are essential in identifying new molecular entities.^9^

**Fig. 1:**
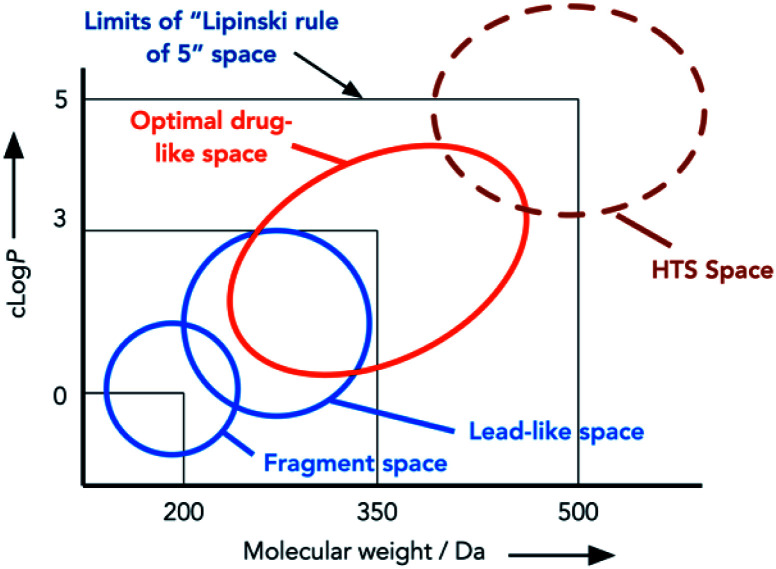
Areas of chemical space targeted by different synthetic approaches. Adapted from Nadin *et al.*^17^

In the late 1990s, Lipinski devised the ‘rule of 5’ (Ro5), a set of criteria that successful small molecule orally available drug compounds often adhere to (Fig. 1).^10^ It has been noted that molecules at the limit of the Ro5 space are not always ideal drug candidates, and screening libraries generated by high-throughput screening (HTS) in the 1990s were not structurally diverse.^11^ Diversity-oriented synthesis (DOS) was developed to focus on creating structural diversity in compound libraries in order to avoid narrow biological activity.^12–14^ DOS involves generating libraries with variation in structure and functionality from simple starting materials *via* complexity incorporating reactions such as cycloadditions.^15^ Some DOS approaches have been used to increase the fraction of sp^3^ hybridization (Fsp^3^) and generate natural product-like screening sets.^16^ But as with HTS, DOS libraries have been criticized for not being ‘lead-like’, that is too large and lipophilic.^17^ The notion of lead-oriented synthesis (LOS) was developed to focus on smaller molecules in lead-like libraries.^17^ LOS is achieved through the application of synthetic methods that deliver libraries with more desirable molecular properties. These libraries can then be further manipulated to afford the diverse substrate sets which have ‘ideal’ physicochemical properties that tend towards the Lipinski space. Libraries of lead-like compounds with the desired properties have been generated by LOS, however this recent development has yet to yield clinical candidates.^18,19^

Fragment-based drug discovery (FBDD) was a term first used around 20 years ago, and has become a significant part of many drug discovery programs since.^20^ It focuses on creating smaller (<20 heavy atoms (not H)), diverse sets of simple molecules which have weak interactions with targets of interest that more likely adhere to a rule of three (Ro3: MW <300 Da, <3 rotatable bonds, cLogP <3).^21,22^ These weak interactions are characterized through biophysical methods (NMR, X-ray crystallography) and this informs the synthetic evolution of the fragment to design the final compound.^23^ The success of FBDD over the last two decades is highlighted by the number of candidates that have reached clinical trials, and even full approval.^20^

A recent essay by Murray and Rees discussed the opportunities and challenges that were presented to synthetic chemists to help with the generation of suitable fragments for FBDD.^24^ The essay stated two requirements of organic synthesis for FBDD:

1. The design and synthesis of new fragments: there is a need for more unique fragments to complement those that are already available.

2. Elaboration of weakly binding fragments to nM leads: a toolbox of synthetic methods should be available to elaborate the fragments upon biological characterization.

An additional key desire was for ‘*methodology that works in the presence of polar groups and permits functionalization via multiple growth positions*’.^24^ In addition, Erlanson *et al.* also highlighted that over the next 20 years ‘*The content of fragment libraries* [may be] *better satisfied by more-3D compounds*’.^20^ The requirement for this type of methodology offers a prime opportunity for biocatalysis.

## Biocatalysis

Biocatalysts are naturally occurring catalysts that operate with high selectivity.^25^ Their inherently selective nature makes enzymes perfect candidates for fragment generation, delivering small, poly-functional molecules without the need for protecting-group chemistry. The number of available biocatalysts has been significantly expanded by the advent of directed evolution in the early 1990s.^26^ Through protein engineering and directed evolution, chemists can now improve enzymes and change their activity, delivering biocatalysts that are not only process-suitable, but also with non-natural substrate tolerance. The chemistry of engineered enzymes now includes C–H hydroxylation,^27^ amine oxidation,^28^ sulfide oxidation,^29^ conjugate reduction,^30^ and sp^2^-halogenation,^31^ with most proceeding with perfect selectivity. This ever-increasing range of reactions underlines the role that biocatalysis could play in the discovery of new biologically active molecules.

There are several reports of enzymes being engineered to address issues in pharmaceutical manufacturing. A landmark example was the directed evolution of a transaminase to replace a Rh-catalysed high-pressure hydrogenation.^32^ The resulting biocatalyst displayed improvements of 13% increase in overall yield, 53% increase in productivity and a 19% reduction in waste produced. Whilst this remarkable process improvement underlined the potential of enzymes for synthetic chemistry, biocatalysis has still yet to see significant application in early stage drug discovery. This is despite the benefits that biocatalysts can impart, namely functionalized, non-protected aliphatic molecules, that have the potential to provide ideal starting points for drug discovery.^33^ A recent article from the Arnold group explored the use of engineered P450 enzymes being used in concert with a Suzuki cross coupling reaction to generate a small library (only four compounds) of cyclopropane derivatives in the context of DOS.^34^

**Scheme 1:**
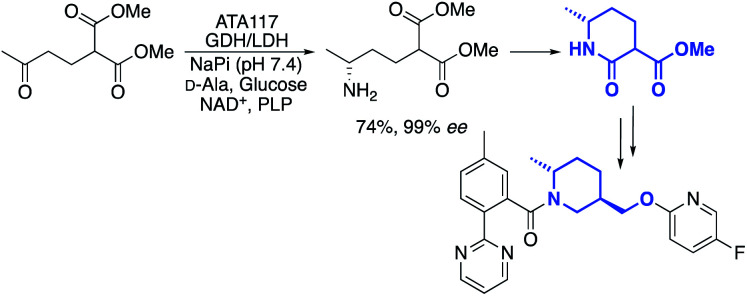
Merck transaminase process for lactam intermediate synthesis.

One example, from the labs of Merck, was a transamination that was used to cyclise a prochiral diester compound to produce a chiral lactam intermediate, used for the synthesis of an API in insomnia treatment (Scheme 1).^35^

**Fig. 2:**
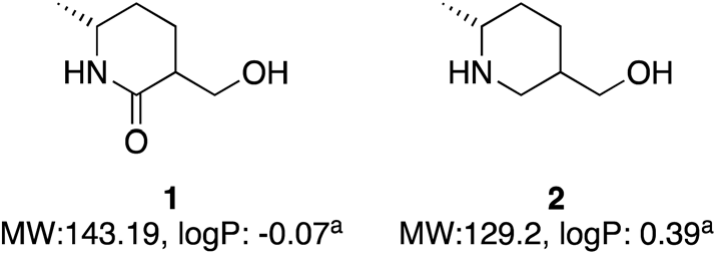
Two molecules with molecular weight and predicted logP values. ^a^logP values calculated at virtual computational chemistry laboratory.^36^

A global or partial reduction could lead to the fragments 1 and 2, which with careful selection of biocatalyst could also generate either enantiomer alpha to nitrogen and therefore deliver diastereomeric control (Fig. 2).

Both molecules contain functional handles for further manipulation, as well as binding functions for activity. They have molecular weights under 200 and predicted logP below 0.5. They also contain multiple C–H bonds with different vectors for growth, so with C–H functionalization one could envisage a host of derivatives that could be synthesized.

Below, we discuss the implications of using biocatalysis for fragment generation, and how it could also play a role in the further functionalization and elaboration of these fragments to for FBDD. Using the open-source computational tool LLAMA (Lead-Likeness and Molecular Analysis)^37^ we assessed the three-dimensionality of biocatalysis products with fragment-like properties, and simulated their elaboration to generate plots detailing where they and their potential derivatives sit within chemical space. LLAMA is capable of assessing candidate molecules as “scaffolds” to be decorated through the simulation of reactions common to the medicinal chemist's toolbox, allowing for evaluation of the lead-likeness of molecules alongside principle moment of inertia (PMI). It was developed with the aim of aiding lead-oriented synthesis, not FBDD, however we re-tasked to the tool to the generation of virtual libraries due to the ease with which it generates predicted molecular properties and virtual libraries of decorated compounds. PMI has been used in a crude way to describe molecular geometry and shape, and is discussed in more detail elsewhere.^38^ We aimed to cover the breadth of the biocatalytic toolbox, with areas such as lone enzyme, biocatalytic cascade and chemoenzymatic synthesis considered through literature highlights. The potential impact of directed evolution on fragment generation, a technology which has hugely advanced the process application of biocatalysts, is also discussed as recent applications of this technique have tooled enzymes towards chemistry not only novel to biocatalysis, but wider synthesis.

## Biocatalysis for fragment synthesis

### Lone-enzyme synthesis of fragment-like molecules

**Scheme 2:**
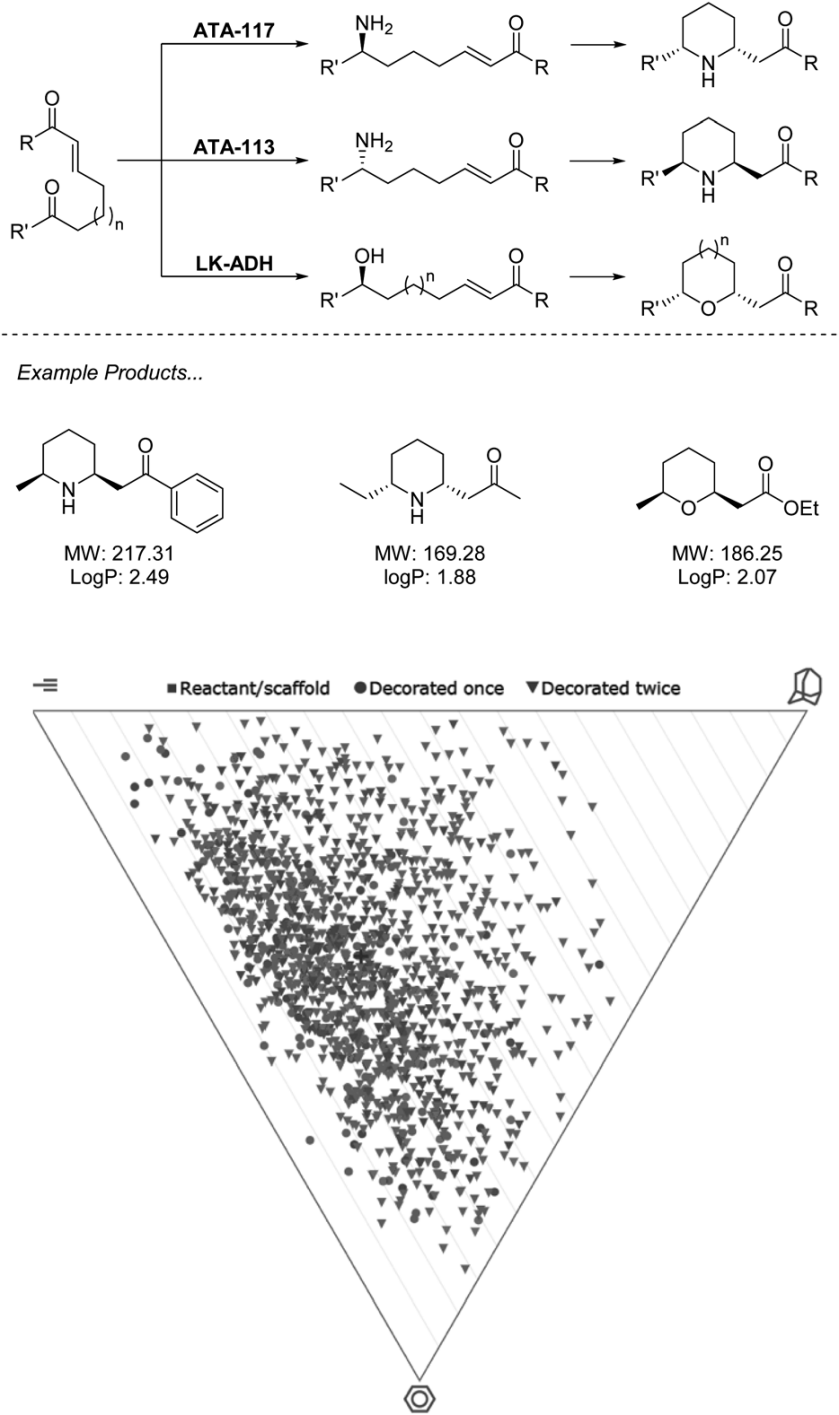
Biocatalytic cascade for generation of *N*- and *O*-heterocycles using ω-TA and ADH. Virtual plots generated using LLAMA software.^37^

While currently underutilized, the ability of biocatalysis to generate a range of complex structures from simple precursors asymmetrically makes it an ideal technology for fragment-like generation. Advantages over chemocatalysts such as chemoselectivity allow for retrosynthetic disconnections not possible with traditional synthesis.^39^ In the example from O'Reilly and co-workers (Scheme 2), both transaminase (TA) and alcohol dehydrogenase (ADH) enzymes are applied for selective transformation of a ketone, which may then be followed by an aza/oxo-Michael epimerization cascade to generate functionalized fragments carrying two stereocenters from simple starting materials.^40,41^

In the first of these two manuscripts, the technique is demonstrated with the stereocomplementary and commercially available TA enzymes “ATA-117” and “ATA-113”.^40^ The exceptional selectivity of these enzymes allows for a retrosynthetic Michael disconnection in which amination exclusively takes place at the ketone carbonyl, and also avoids the amination of the product ketone. This transformation also proceeds entirely stereoselectively, with an *ee* of >99%, which directs the proceeding epimerization and retains the asymmetry. The authors demonstrate that through the choice of enzyme, either enantiomer of product may be accessed with absolute selectivity. While many chemocatalytic methodologies may deliver enantiomerically enriched products, the unparalleled stereoselectivity that results from the environment of an enzyme active site presents a huge advantage to fragment generation in which architectures are constructed to probe an asymmetric environment and therefore require stereopurity for true evaluation. This methodology has recently seen expansion to alkyne equivalents, generating exo-cyclic enamines.^42^

In the follow-up manuscript to this work, O'Reilly and co-workers again demonstrate how enzyme selectivity may be exploited to perform this challenging retrosynthetic disconnection through the use of an ADH to deliver a range of tetrahydropyrans and tetrahydrofurans, moieties that are both challenging to incorporate into synthesis and readily present in bioactive molecules.^41^ Analysis of the products described across the two manuscripts resulting from this biocatalytic methodology reveals that the heterocycles obtained present an opportunity to cover a wide region of chemical space. Example products are within the parameters of the Ro3 and may be functionalised along multiple vectors to yield diverse fragments. Reduction of the carbonyls in the final products could be easily achieved biocatalytically or chemically, or hydrolysis of the ester to produce the acid, thus removing the undesirable functionality and still producing fragments in minimum synthetic operations.

With the biocatalysts from this work having been sourced commercially and applied on a preparative scale, this methodology highlights a prime example of the opportunity that biocatalysis offers to generate a diverse library of asymmetric sp^3^-rich fragments from simple precursors (Scheme 2).

Whilst the excellent selectivity of biocatalysis is indicative of the power of this technology for controlled building into novel chemical space, the toolbox remains limited to a basic set of reactions when compared to those achievable through the use of wider synthetic methods. To combat this, a strategy of chemoenzymatic synthesis may be explored in which an enzyme may select initial stereochemistry before products that would be inaccessible using known biocatalytic techniques are synthesized by methods that retain configuration.

### Chemoenzymatic synthesis of azepanes

**Scheme 3:**
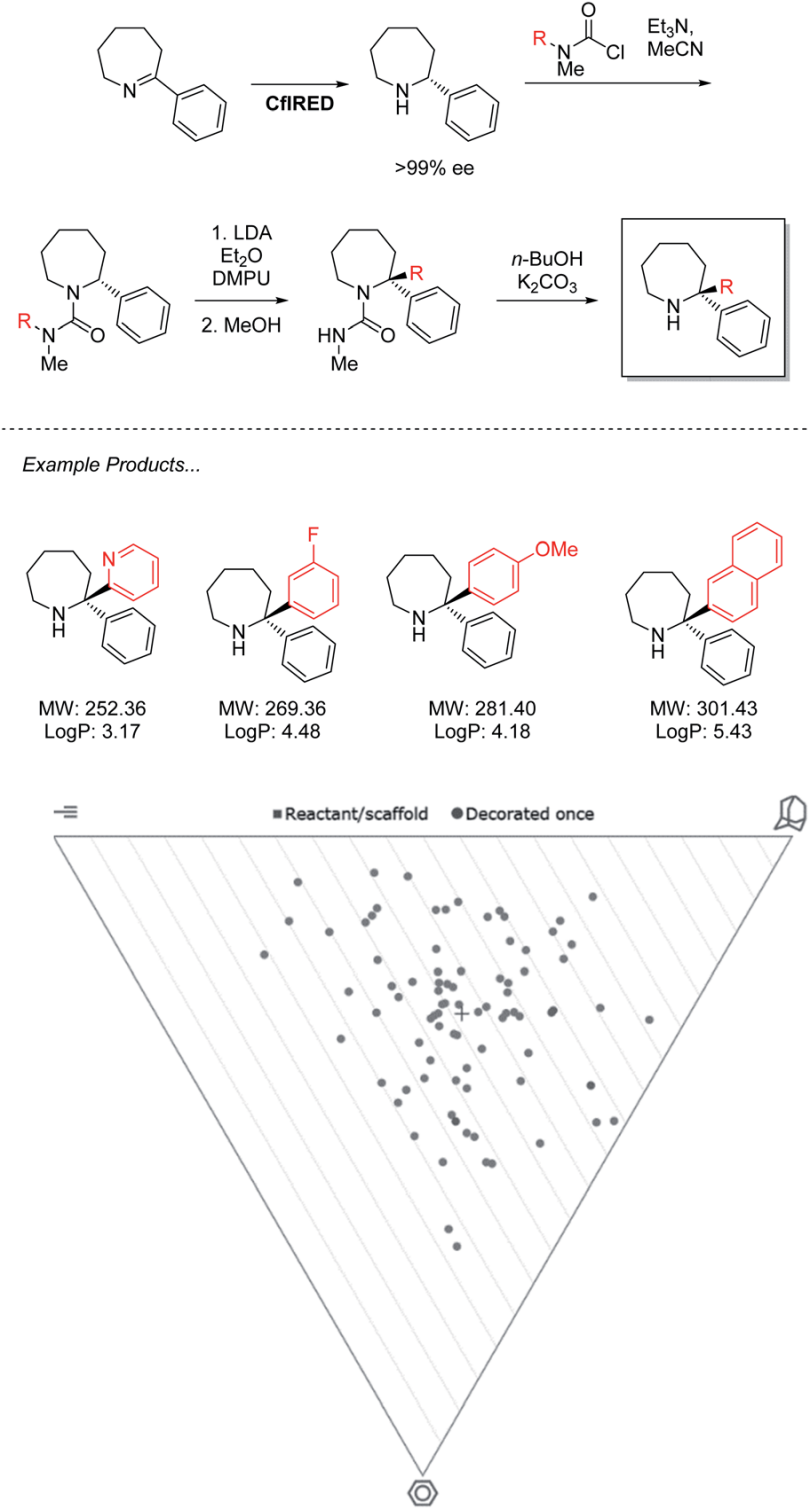
IRED reduction/lithiation chemoenzymatic synthesis of 2,2-disubstituted azepane derivatives.

The power of this approach to access challenging architectures has been demonstrated by collaboration between the groups of Turner & Clayden (Scheme 3).^43^ Amine oxidases and imine reductases (IREDs) are well established enzymes for the chiral resolution of saturated amine heterocycles, with certain members of the latter family demonstrating a further ability to catalyse reductive amination.^44,45^ This gives rise to biocatalytic strategies for the synthesis of asymmetric azepanes, a biologically relevant yet underrepresented class of heterocycle.

Despite enantiocomplementary methods that proceed with full selectivity, the reaction itself is a simple reduction that may only generate products with a singular substitution at the α carbon.

To access 2,2-disubstituted products the authors envisaged a strategy of conversion to the corresponding *N*′-arylated urea compounds followed by a lithium-mediated transfer of said aryl substituent to the 2-position of the azepane and finally, deprotection. Using this methodology a large product scope of 2,2-diarylated azepane scaffolds is generated by the authors with medicinal relevance speculated. Analysis of these products reveals a library of compounds that heavily inhabit “sphere-like” chemical space. As molecules of this shape are the most underexplored in FBDD, a strong potential for novel bioactivity is implied. However, the products are limited in fragment-like characteristics due to the lipophilicity of the diarylated products that lack functional handles. For this methodology to find application in medicinal chemistry, functional limitations must be understood, and expansion should be sought to incorporate further heteroatoms into both saturated and aromatic rings. With the timescale pressures brought on by the economic requirements of a pharmaceutical campaign, the need to optimize both concurrent bio and chemocatalytic steps presents a serious challenge. This underlines a requirement for the development of more facile biocatalytic methodology to facilitate its adaptation in medicinal chemistry.

### Engineered metalloenzyme cyclopropanation

**Scheme 4:**
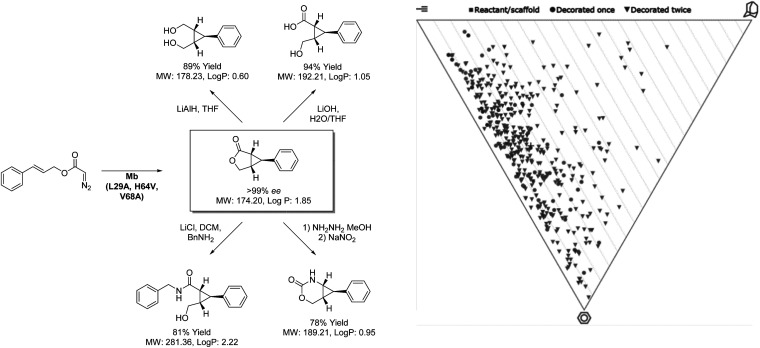
Intramolecular cyclopropanation catalysed by myoglobin and coupled transformation of product.

While difficulties in the optimization of chemoenzymatic synthesis may limit its application, directed evolution allows for an alternative approach to expanding the biocatalytic toolbox beyond the scope of nature. As a technique, directed evolution has been paramount in the success of biocatalytic process chemistry with virtually all commercially applied enzymes having been subjected to rounds of optimization. Whilst the technique is typically considered as a method of improving enzyme stability and substrate scope, it is increasingly being applied by researchers to develop enzymes capable of performing new transformations, often not only novel to biocatalysis but synthesis as a whole. This was demonstrated to great effect by Fasan and co-workers (Scheme 4) in the development of an engineered biocatalyst from sperm whale myoglobin capable of catalysing intramolecular cyclopropanation.^46^ Whilst this transformation has been demonstrated by chemocatalytic methods, performing this complex rearrangement within the confines of an enzyme active site presents a unique challenge. However, through separate directed evolution campaigns a toolbox of stereocomplementary carbene transferases were created from this oxygen storage protein. Remarkably, one (1*R*,5*S*,6*S*)-selective variant was noted to reach 74% conversion to product within 15 minutes, with an enantiopurity of 99% when applied as a whole cell biocatalyst. The ability of this catalyst to generate 3 chiral centers from symmetrical starting materials with complete stereocontrol highlights a huge potential for this biocatalyst in providing fragment architectures. Furthermore, the outstanding reaction rates provided by the closed active site and the ease of accessing the catalyst through simply culturing *E. coli* demonstrates the strong economic potential of introducing biocatalysis to medicinal chemistry. While analysis reveals potential to build into three-dimensional space, many compounds generated from this product scope tended towards flatness on the PMI plot. This is likely related to the limitations of LLAMA as software, which is unable to cleave the initially formed lactone or add to resulting functionalities. Despite this, these unique scaffolds all display fragment-like properties. Through further manipulation of the products shown, more highly saturated fragments may be envisaged, through biocatalytic means or otherwise. In addition to the products shown there, the Fasan group has extended the scope of the methodology to include fused cyclopropyl-benzofurans and lactams.^47,48^ The diverse nature of the products generated from these studies highlight the direction that biocatalysis will take in the coming years, producing scaffolds with increasing complexity through less synthetic steps than is possible with other methods.

### Biocatalysis for fragment elaboration

As mentioned earlier, as well as fragment synthesis, another important role for chemistry in FBDD is to be able to provide methods for further functionalization of fragments.^24^ It is clear, especially given the pace of growth of C–H activation methodology and photoredox catalysis for example,^49,50^ that many transformations could be applied to the fragments shown above. Indeed, C–H functionalization methodology as a tool for direct elaboration of hits has been discussed and reported recently.^51^ There is further potential for biocatalysis here as well, with functionalization of inert C–H bonds attainable with several routinely used enzymes.

### Engineered P450s

The cytochrome P450 enzyme class is well known for its importance in metabolism, being used to identify potential metabolites of drug candidates.^52^ In biocatalysis, further application has been demonstrated in the mediation of synthetic transformations.^53,54^ Despite being widely and well known as C–H hydroxylation catalysts, recent engineering efforts from several groups, but pioneered by the Arnold group, have expanded the scope of the P450 toolbox.^55^

**Scheme 5:**
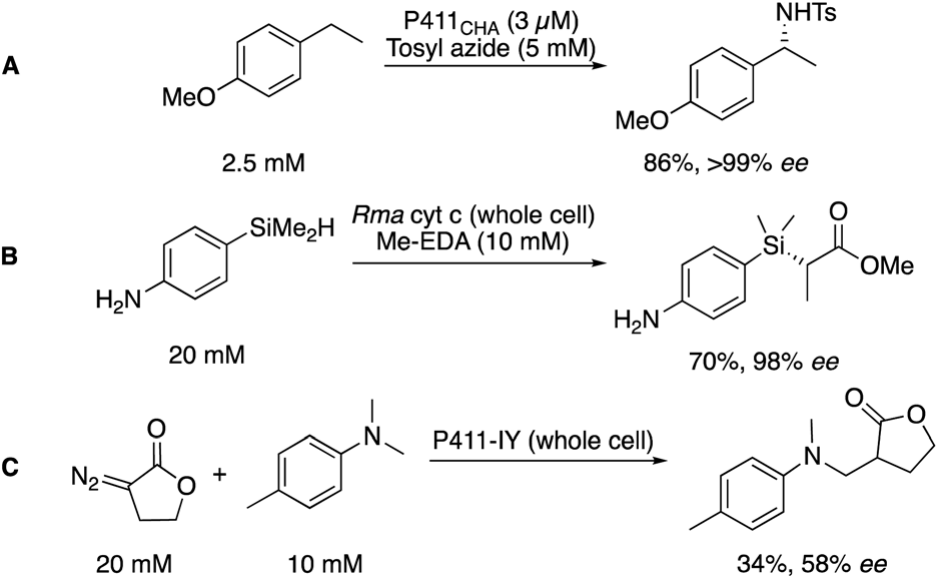
C–H activation chemistry attainable with engineered P411 biocatalysts.

Arnold and co-workers found that a single mutation of the P450 active site could alter the specificity of P450s, shifting a signature UV-Vis peak for the CO-bound complex from 450 to 411 nm, and dubbing this new, engineered class of biocatalysts the P411.^56^ Importantly, this new enzyme had a higher affinity for the transfer of complexed carbenes as opposed to oxygen, generating an engineered biocatalyst capable of mediating the cyclopropanation of alkenes (similar to that discussed in Scheme 4). This has since been expanded to encompass functionalization of C–H bonds with a myriad of nucleophilic partners for formal C–H amination (A),^57^ Si–H insertion (B),^58^ and C–C bond formation (C, Scheme 5).^59^

The significance of the P411 toolbox, as well as other similar engineered heme enzymes,^60^ is that it can be readily expanded through protein engineering to meet to the challenges set by chemists. Furthermore, the freedom to operate in the presence of polar functionality with perfect chemoselectivity is key to solving the problems presented to chemists in fragment elaboration.^20,24^

### Halogenases

Another class of enzyme key to elaboration success are the halogenases.^31^ Halogenase biocatalysts transform inert C–H bonds, primarily sp^2^, to an organohalide bond, proceeding under ambient conditions using inorganic halide sources. According to Roughley and Jordan's analysis, 20% of medicinal chemistry reactions involve the use, or synthesis of organohalides, underlining the importance of methods for their production.^5^ Therefore, the ability to perform this with enzymes presents an opportunity to overcome a key limitation of FBDD: the selective functionalization of identified fragments allows for new strategies for elaboration that do not disrupt the binding motif.

**Scheme 6:**
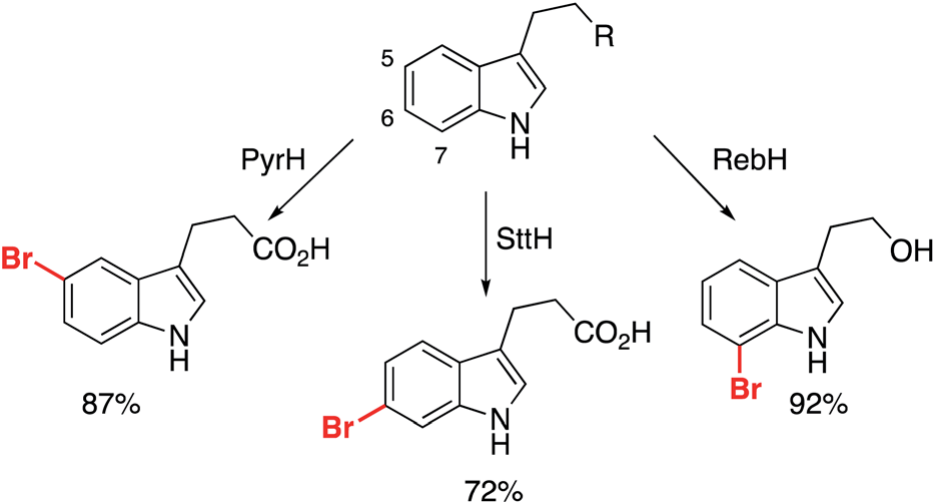
Different halogenases permitting the selective bromination of non-protected indole derivatives.

Several reports have detailed two-step procedures whereby *in situ* halogenation is followed by functionalization of the activated bond. One example from the Lewis group showed bromination of several indole derivatives, which was coupled to Pd-catalysed transformations of the C–Br bond.^61^ It was achieved with work-up and then exposure of the crude biotransformation procedure to the Pd-catalyst preparation. The Micklefield and Greaney labs overcame the incompatibility issues through the use of a siloxane membrane, but importantly two of the halogenases used permitted functionalization of indole skeletons at the 5- and 6- positions, transformations which are challenging for traditional synthetic catalysts (Scheme 6).^62^

These substrates were all subsequently converted to the respective arylated derivatives in one-pot, demonstrating the applicability of the reported method.^62^ Importantly, it demonstrates that growth of these fragments can be achieved into multiple vectors without the need for protection of the parent scaffold (free N–H/O–H/CO_2_H), and without interrupting the binding modes of the molecules. Further work is required to discover new halogenase enzymes to expand the substrate scope. An elegant example from Goss and co-workers recently revealed the first natural iodinase, which had a broad substrate scope towards the iodination of (hetero)aromatics.^63^ A genome mining approach to biocatalyst discovery uncovered new sequences that were distinct from many of the reported halogenases to date. This approach, combined with protein engineering, could open up the application of halogenases and make them essential tools for late-stage functionalization.

### Future of biocatalysis for FBDD

#### Protein discovery and engineering

Biocatalysis could clearly play a role in the future of fragment generation and elaboration, addressing two of the main challenges set to synthetic chemists by medicinal chemists.^20,24^ There are still many areas in which biocatalysis must improve to fully realise its potential, not only in FBDD but wider synthesis.

The scope of non-natural substrates must be significantly increased to allow off-the-shelf biocatalyst application. Genome mining approaches, such as that from the Goss group for iodinase discovery,^63^ will certainly aid this, as uncovering new sequences and proteins will uncover many undiscovered ‘natural’ chemistries. Additionally, expansion of the genetic code to incorporate non-canonical amino acids can help increase the chemistry that enzymes can do. A recent study by Green and co-workers showed that incorporation of non-canonical amino acid *N*-methylhistidine could accelerate the rate of reaction of an artificial hydrolytic enzyme (BH32), with the final mutant described in the study showing >9000 fold improvement in activity *versus N*-methylhistidine in solution.^64^ This remarkable study shows what computational power and HTS can do for the design of new biocatalysts.

Concomitantly to new enzyme discovery and design, there needs to be comprehensive protein engineering campaigns to further improve the stability and activity of novel biocatalysts. Being able to easily apply them under industry-ready conditions is essential to their combination with synthetic catalysts in other applications.

#### Reaction engineering

Alongside protein engineering, reaction engineering and process optimization solutions can also change the way that biocatalysts are applied in the synthesis of small molecules. Flow chemistry has emerged as an important tool for organic synthesis in the past 20 years,^65^ and this has recently become more prominent in biocatalysis as well.^66,67^ The use of flow chemistry for biocatalysis continues to offer solutions to problems that have proven long-standing and hard to overcome, such as enzyme stability with immobilization,^68^ improving the kinetics of an enzymatic reaction by novel reactor designs,^69^ and the easier integration of organic solvents.^70^ Other technological solutions, including the use of non-aqueous medias such as deep eutectic solvents (DESs),^71^ or membrane separation for reaction compartmentalization,^72^ provide a glimpse as to some of the potential ways biocatalysts could be applied to circumvent issues with their application.

Importantly, the use of new technologies, such as compartmentalization or flow chemistry, will allow greater ease of integration with more traditional synthetic methods. This synergy between traditional and biocatalytic techniques, which is already well documented,^73^ will streamline application of biocatalysis in synthetic routes.

## Conclusions

Biocatalysis continues to evolve at a fast pace and as a consequence the way we can use it, and envisage using it, is changing rapidly. Already established as a key tool in process development in the pharmaceutical industry, the diversity of new chemistry that can now be performed with enzymes presents an opportunity for increased application in early-stage fragment-based drug discovery programs. The scope of the biocatalytic toolbox is ever expanding, and with new technological solutions being presented with equal pace, the opportunity for integration into fragment generation programs is greater than ever.

## Conflicts of interest

There are no conflicts to declare.

## References

[cit1] Walters W. P., Green J., Weiss J. R., Murcko M. A. (2011). J. Med. Chem..

[cit2] Wang J., Hou T. (2010). J. Chem. Inf. Model..

[cit3] Taylor R. D., MacCoss M., Lawson A. D. G. (2017). J. Med. Chem..

[cit4] Boström J., Brown D. G., Young R. J., Keserü G. M. (2018). Nat. Rev. Drug Discovery.

[cit5] Roughley S. D., Jordan A. M. (2011). J. Med. Chem..

[cit6] Brown D. G., Boström J. (2016). J. Med. Chem..

[cit7] Lovering F., Bikker J., Humblet C. (2009). J. Med. Chem..

[cit8] Karawajczyk A., Giordanetto F., Benningshof J., Hamza D., Kalliokoski T., Pouwer K., Morgentin R., Nelson A., Müller G., Piechot A., Tzalis D. (2015). Drug Discovery Today.

[cit9] Foley D. J., Nelson A., Marsden S. P. (2016). Angew. Chem., Int. Ed..

[cit10] Lipinski C. A., Lombardo F., Dominy B. W., Feeney P. J. (1997). Adv. Drug Delivery Rev..

[cit11] Grygorenko O. O., Volochnyuk D. M., Ryabukhin S. V., Judd D. B. (2019). Chem. – Eur. J..

[cit12] Schreiber S. L. (2000). Science.

[cit13] Galloway W. R. J. D., Isidro-Llobet A., Spring D. R. (2010). Nat. Commun..

[cit14] Trabocchi A. , Diversity-Oriented Synthesis: Basics and Applications in Organic Synthesis, Drug Discovery, and Chemical Biology, John Wiley & Sons Ltd, New Jersey, 2013

[cit15] Gerry C. J., Schreiber S. L. (2020). Curr. Opin. Chem. Biol..

[cit16] Morton D., Leach S., Cordier C., Warriner S., Nelson A. (2009). Angew. Chem., Int. Ed..

[cit17] Nadin A., Hattotuwagama C., Churcher I. (2012). Angew. Chem., Int. Ed..

[cit18] Doveston R. G., Tosatti P., Dow M., Foley D. J., Li H. Y., Campbell A. J., House D., Churcher I., Marsden S. P., Nelson A. (2015). Org. Biomol. Chem..

[cit19] Foley D. J., Doveston R. G., Churcher I., Nelson A., Marsden S. P. (2015). Chem. Commun..

[cit20] Erlanson D. A., Fesik S. W., Hubbard R. E., Jahnke W., Jhoti H. (2016). Nat. Rev. Drug Discovery.

[cit21] Congreve M., Carr R., Murray C., Jhoti H. (2003). Drug Discovery Today.

[cit22] Jhoti H., Williams G., Rees D. C., Murray C. W. (2013). Nat. Rev. Drug Discovery.

[cit23] Erlanson D. A., Davis B. J., Jahnke W. (2019). Cell Chem. Biol..

[cit24] Murray C. W., Rees D. C. (2016). Angew. Chem., Int. Ed..

[cit25] Sheldon R. A., Brady D., Bode M. L. (2020). Chem. Sci..

[cit26] Arnold F. H. (2018). Angew. Chem., Int. Ed..

[cit27] Bernhardt R., Urlacher V. B. (2014). Appl. Microbiol. Biotechnol..

[cit28] Turner N. J. (2011). Chem. Rev..

[cit29] Bong Y. K., Song S., Nazor J., Vogel M., Widegren M., Smith D., Collier S. J., Wilson R., Palanivel S. M., Narayanaswamy K., Mijts B., Clay M. D., Fong R., Colbeck J., Appaswami A., Muley S., Zhu J., Zhang X., Liang J., Entwistle D. (2018). J. Org. Chem..

[cit30] Toogood H. S., Scrutton N. S. (2018). ACS Catal..

[cit31] Latham J., Brandenburger E., Shepherd S. A., Menon B. R. K., Micklefield J. (2018). Chem. Rev..

[cit32] Savile C. K., Janey J. M., Mundorff E. C., Moore J. C., Tam S., Jarvis W. R., Colbeck J. C., Krebber A., Fleitz F. J., Brands J., Devine P. N., Huisman G. W., Hughes G. J. (2010). Science.

[cit33] Devine P. N., Howard R. M., Kumar R., Thompson M. P., Truppo M. D., Turner N. J. (2018). Nat. Rev. Chem..

[cit34] Wittmann B. J., Knight A. M., Hofstra J., Reisman S. E., Kan S. B. J., Arnold F. H. (2020). ACS Catal..

[cit35] Chung J. Y. L., Marcune B., Strotman H. R., Petrova R. I., Moore J. C., Dormer P. G. (2015). Org. Process Res. Dev..

[cit36] Tetko I. V., Gasteiger J., Todeschini R., Mauri A., Livingstone D., Ertl P., Palyulin V. A., Radchenko E. V., Zefirov N. S., Makarenko A. S., Tanchuk V. Y., Prokopenko V. V. (2005). J. Comput.-Aided Mol. Des..

[cit37] Colomer I., Empson C. J., Craven P., Owen Z., Doveston R. G., Churcher I., Marsden S. P., Nelson A. (2016). Chem. Commun..

[cit38] Sauer W. H. B., Schwarz M. K. (2003). J. Chem. Inf. Comput. Sci..

[cit39] Turner N. J., O'Reilly E. (2013). Nat. Chem. Biol..

[cit40] Ryan J., Šiaučiulis M., Gomm A., Maciá B., O'Reilly E., Caprio V. (2016). J. Am. Chem. Soc..

[cit41] Eastman H., Ryan J., Maciá B., Caprio V., O'Reilly E. (2019). ChemCatChem.

[cit42] Taday F., Ryan J., Argent S. P., Caprio V., Maciá B., O'Reilly E. (2020). Chem. – Eur. J..

[cit43] Zawodny W., Montgomery S. L., Marshall J. R., Finnigan J. D., Turner N. J., Clayden J. (2018). J. Am. Chem. Soc..

[cit44] Mangas-Sanchez J., France S. P., Montgomery S. L., Aleku G. A., Man H., Sharma M., Ramsden J. I., Grogan G., Turner N. J. (2017). Curr. Opin. Chem. Biol..

[cit45] Batista V. F., Galman J. L., Pinto D. C., Silva A. M. S., Turner N. J. (2018). ACS Catal..

[cit46] Chandgude A. L., Ren X., Fasan R. (2019). J. Am. Chem. Soc..

[cit47] Vargas D. A., Khade R. L., Zhang Y., Fasan R. (2019). Angew. Chem., Int. Ed..

[cit48] Ren X., Chandgude A. L., Fasan R. (2020). ACS Catal..

[cit49] Shaw M. H., Twilton J., MacMillan D. W. C. (2016). J. Org. Chem..

[cit50] Crabtree R. H., Lei A. (2017). Chem. Rev..

[cit51] Grainger R., Heightman T. D., Ley S. V., Lima F., Johnson C. N. (2019). Chem. Sci..

[cit52] Rendic S., Guengerich F. P. (2015). Chem. Res. Toxicol..

[cit53] Whitehouse C. J. C., Bell S. G., Wong L. L. (2012). Chem. Soc. Rev..

[cit54] Roiban G. D., Reetz M. T. (2015). Chem. Commun..

[cit55] Brandenberg O. F., Fasan R., Arnold F. H. (2017). Curr. Opin. Biotechnol..

[cit56] Coelho P. S., Wang Z. J., Ener M. E., Baril S. A., Kannan A., Arnold F. H., Brustad E. M. (2013). Nat. Chem. Biol..

[cit57] Prier C. K., Zhang R. K., Buller A. R., Brinkmann-Chen S., Arnold F. H. (2017). Nat. Chem..

[cit58] Kan S. B. J., Lewis R. D., Chen K., Arnold F. H. (2016). Science.

[cit59] Zhang R. K., Chen K., Huang X., Wohlschlager L., Renata H., Arnold F. H. (2019). Nature.

[cit60] Zhang R. K., Huang X., Arnold F. H. (2019). Curr. Opin. Chem. Biol..

[cit61] Durak L. J., Payne J. T., Lewis J. C. (2016). ACS Catal..

[cit62] Latham J., Henry J. M., Sharif H. H., Menon B. R. K., Shepherd S. A., Greaney M. F., Micklefield J. (2016). Nat. Commun..

[cit63] Gkotsi D. S., Ludewig H., Sharma S. V., Connolly J. A., Dhaliwal J., Wang Y., Unsworth W. P., Taylor R. J. K., McLachlan M. M. W., Shanahan S., Naismith J. H., Goss R. J. M. (2019). Nat. Chem..

[cit64] Burke A. J., Lovelock S. L., Frese A., Crawshaw R., Ortmayer M., Dunstan M., Levy C., Green A. P. (2019). Nature.

[cit65] Plutschack M. B., Pieber B., Gilmore K., Seeberger P. H. (2017). Chem. Rev..

[cit66] Britton J., Majumdar S., Weiss G. A. (2018). Chem. Soc. Rev..

[cit67] Thompson M. P., Peñafiel I., Cosgrove S. C., Turner N. J. (2019). Org. Process Res. Dev..

[cit68] Romero-Fernández M., Paradisi F. (2020). Curr. Opin. Chem. Biol..

[cit69] Chapman M. R., Cosgrove S. C., Turner N. J., Kapur N., Blacker A. J. (2018). Angew. Chem., Int. Ed..

[cit70] Böhmer W., Volkov A., Engelmark Cassimjee K., Mutti F. G. (2020). Adv. Synth. Catal..

[cit71] Cicco L., Ríos-Lombardía N., Rodríguez-Álvarez M. J., Morís F., Perna F. M., Capriati V., García-Álvarez J., González-Sabín J. (2018). Green Chem..

[cit72] Sato H., Hummel W., Gröger H. (2015). Angew. Chem., Int. Ed..

[cit73] Huang X., Cao M., Zhao H. (2020). Curr. Opin. Chem. Biol..

